# The design of a community lifestyle programme to improve the physical and psychological well-being of pregnant women with a BMI of 30 kg/m^2 ^or more

**DOI:** 10.1186/1471-2458-10-284

**Published:** 2010-05-27

**Authors:** Debbie M Smith, Melissa Whitworth, Colin Sibley, Wendy Taylor, Jane Gething, Catherine Chmiel, Tina Lavender

**Affiliations:** 1 The School of Nursing, Midwifery and Social Work, The University of Manchester, Jean MacFarlane Building, Oxford Road, Manchester, M13 9PL, UK; 2 Department of Obstetrics, St Mary's Hospital, Manchester, UK; 3 Maternal & Fetal Health Research Centre, Research School of Clinical & Laboratory Sciences, The University of Manchester, St Mary's Hospital, Manchester, UK

## Abstract

**Background:**

Obesity is a global public health issue. Having a BMI of 30 kg/m^2 ^or more (classifying a person as obese) at the start of pregnancy is a significant risk factor for maternal and fetal morbidity. There is a dearth of evidence to inform suitable inteventions to support pregnant women with a BMI of 30 kg/m^2 ^or more. Here we describe a study protocol to test the feasibility of a variety of potential healthy lifestyle interventions for pregnant women with a BMI of 30 kg/m^2 ^or more in a community based programme.

**Methods/Design:**

Four hundred women will be approached to attend a 10-week community lifestyle programme. The programme will be provided as a supplement to standard antenatal care. The programme is multi-faceted, aimed at equipping participants with the skills and knowledge needed to adopt healthy behaviours. The social (cognitive) learning theory will be used as a tool to encourage behaviour change, the behaviour change techniques are underpinned by five theoretical components; self-efficacy, outcome expectancies, goal setting, feedback and positive reinforcement.

The main outcomes are pregnancy weight gain and caesarean section rate. Other important outcomes include clinical outcomes (e.g., birth weight) and psychological outcomes (e.g., well-being). Secondary outcomes include women's experience of pregnancy and health care services, amount of physical activity, food intake and the suitability of the intervention components.

A prospective study using quantitative and qualitative methods will inform the feasibility of implementing the community lifestyle programme with pregnant women with a BMI of 30 kg/m^2 ^or more. Mixed methods of data collection will be used, including diaries, focus groups/interviews, pedometers, validated and specifically designed questionnaires, a programme register, weight gain during pregnancy and perinatal outcome data.

**Discussion:**

Findings from this current feasibility study will inform future interventions and NHS services and add to the evidence-base by providing information about the experiences of pregnant women with a BMI of 30 kg/m^2 ^or more undertaking a community lifestyle programme. The study will lead on to a randomised control trial of a suitable intervention to improve the pregnancy outcomes of this target group.

**Trail Registration:**

ISRCTN29860479.

## Background

A body mass index (BMI) of 30 kg/m^2 ^or more is classified clinically as obese, a BMI of 35 or more is morbidly obesity and a BMI of 40 or more indicates extreme obesity (World Health Organisation, 1998). Obesity is a global epidemic [1] and is high priority for Governments around the world. For example, in the United States the prevalence rate of obesity for over 20 year olds was 34% in 2005-2006 [2]. In England, two-thirds of adults and one-third of children are either overweight or obese and predictions suggest this could rise to almost nine in ten adults and two-thirds of children by 2050 [3].

Research from around the world has highlighted that maternal obesity carries significant risks to both mother [e.g. [4,5]] and baby [e.g. [6-8]]. In the US, the percentage of women who are overweight (BMI of 26 or more) at the pre-pregnancy stage has been increasing for over 20 years [9]. In one area of England, the percentage of pregnant women who have a BMI of 30 kg/m^2 ^or more at conception increased from 9% to 16% over a 15-year period [10]. The impact of BMI on pregnancy outcomes in the UK was highlighted in the recent triennium review into maternal mortality highlighted maternal obesity as an 'obstetric risk factor'; 27% of women who died from direct and indirect causes in the UK had a BMI of 30 kg/m^2 ^or more [11].

Excessive weight gain has been found to increase the risk of complications in pregnancy and a low weight gain during pregnancy has been suggested as beneficial for obese women [12]. The Institute of Medicine (IOM) provides ranges of recommended ranges of weight gain for women during pregnancy. These recommended ranges are dependent on the woman's weight at the start of the pregnancy and were revised in 2009. Women with a BMI of more than 30 kg/m^2 ^should not exceed a weight gain of 5-9 kg (the 1990 report stated 7 kgs) during their pregnancy [13]. The IOM's recommended weight-gain ranges have been found to be associated with the best outcomes of pregnancy for mothers in the USA [14]. However, in the UK there is little evidence to support these weight gain recommendations and no guidelines are in place.

As a consequence of this association with adverse outcomes of pregnancy, we are keen to conduct research to improve the health of pregnant women who have a BMI of 30 kg/m^2 ^or more. Although pregnancy has been suggested as an optimal time for intervention by health care professionals involving health promotion messages [15] and national recommendations have suggested healthy pregnancy advice should be provided before 12 weeks gestation [e.g. [16]]. Little guidance is provided for health care professionals for work with this group (NICE guidelines are expected in mid-2010) and as a result there is minimal support, care pathways and services for pregnant women who have a BMI of 30 kg/m^2 ^or more. A systematic review of weight maintenance interventions concluded there is minimal evidence to inform maternal obesity strategies [17].

There is insufficient evidence to inform the design of an effective and appropriate intervention for pregnant women with a BMI of 30 kg/m^2 ^or more as there is a dearth of evidence--based and theory-based research on this target group. The recent NICE commissioned evidence reviews of dietary and/or physical activity interventions for weight management in pregnancy [18] and post-partum [19] found a small number of robust studies in the UK. They concluded that there is 'inconsistent and inconclusive evidence' on the effectiveness of weight management interventions in pregnancy (p.12 [18]).

A recent review by the Kings Fund highlighted several theories of behaviour change (e.g. stages of change model), psychological constructs (e.g. self-efficacy) and types of interventions (e.g. goal-setting) that have been successfully applied to the up-take of physical activity and eating a healthy diet in the general population [20]. The need for community and individualised intervention studies for weight maintence in pregnancy and post-partum have been the conclusions of several studies [21,22] and approaches to lifestyle and behaviour change which ignore the wider social context are likely to have limited success [23,24]. Lifestyle programmes and structured physical activity programmes have equally been found to increase health benefits in healthy sedentary adults [e.g. [25]]. Therefore, it is important when addressing obesity to target numerous health behaviours that contribute to weight gain; physical activity, healthy eating and emotional well-being. In addition, interventions must have theoretical underpinnings such as those listed in the Kings Fund report.

This paper outlines the design (rationale and theoretical-framework) of a community lifestyle programme study which may offer benefit to pregnant women with a BMI of 30 kg/m^2 ^or more by improving their own and their babies' physical and psychological well-being. Our community lifestyle programme will address numerous behaviours associated with obesity (e.g. physical activity, emotional well-being and food-intake) and will equip participants with the necessary skills and knowledge to incorporate healthy behaviours into their everyday lives (social (cognitive) learning theory [e.g. [26]] will be used as a tool to encourage behaviour change [27]). The feasibility of this lifestyle programme will be evaluated so as to assist researchers in the design of future studies and programmes and may be subjected to a trial at a later date.

## Methods/Design

This is a prospective study, using mixed-methods of data collection to inform the feasibility of implementing a community lifestyle programme with pregnant women with a BMI or 30 kg/m^2 ^or more in the Greater Manchester areas of Bolton and Oldham. Randomised controlled trials are considered the gold standard when assessing interventions [28]. However, one has to be sure that the intervention is of the highest standard, and acceptable to those who will be receiving it prior to proceeding to a randomised controlled trial. There is currently no evidence suggesting the optimum intervention for this group of women, nor is there any evidence of intervention acceptability. Therefore, following the advice of the Medical Research Council (MRC) framework for a complex intervention [29] we believed it imperative that a feasibility study was conducted prior to any such trial. Our approach to programme development is one which allows fluidity, i.e. the programme will be adapted contemporaneously following feedback and review.

### Research team

The core research team consists of the principal investigator (Professor of Midwifery), project manager (health psychologist) and two research midwives. An Advisory Board of leading experts (clinicians and academics) in obesity and pregnancy meet twice a year during the study. A Lay Advisory Group; four pregnant women with a BMI or 30 kg/m^2 ^or more in Bolton and four pregnant women with a BMI or 30 kg/m^2 ^or more in Oldham, will meet three times throughout the first programme.

### Setting

The study is being conducted in England, in the Greater Manchester areas of Bolton and Oldham. The two areas have similar ethnic diversity (predominantly white; Bolton - 90% and Oldham - 86% with minority ethnic groups concentrated in small areas), level of health (lower than the National average), deprivation (among the most deprived areas in England) and pregnancy data (maternal obesity is found in approximately 16% of the women delivering) [30].

### Sample

Four hundred pregnant women with a BMI of 30 kg/m^2 ^or more will be approached to participate in the study (200 from each area). Women will be excluded from participation in the study if they have a BMI of less than 30 kg/m^2^, are aged under 18, intend to move in the next three months, take weight control medication or if they have any cautions for starting exercise (this will be determined using the Revised Physical Activity Readiness Questionnaire (PARQ) [31]) and the Royal College of Obstetricians and Gynaecologists (RCOG) recommendations [32]). Their obstetrician will be consulted in cases where the PARQ or RCOG guidelines indicate concerns. Each participant's General Practitioner (GP) will be sent a letter outlining the involvement in the study. The sample size is determined pragmatically by the need to gain sufficient experience of the intervention over a range of settings and participants and thus test feasibility on a realistic basis. 200 participants is more than adequate to estimate standard deviations and 10-12 groups will allow us to estimate the intraclass correlation for the design of future studies. Formal comparisons are pre-post measures where 200 participants will give 80% power to detect small changes of 0.2 or larger, expressed as standardised effect sizes in terms of the intra-patient standard deviation (80% power, paired t-test). The major obstetric outcomes (caesarean section and low birth weight) have expected incidence rates of around 20% [6] and will be estimated with a 95% confidence interval width of approximately 5%, allowing large effects to be detected in comparison with audit data.

### Outcomes

The main outcome of interest is pregnancy weight gain. Other important clinical outcomes include birth weight, mode of birth and method of infant feeding at hospital discharge. Important psychological outcomes include self-efficacy, well-being and goal attainment. Secondary outcomes include women's experience of pregnancy and health care services, amount of physical activity, food intake and the suitability of the intervention components.

### Recruitment

Approval has been gained from the NHS research ethics committee, the individual Primary Care Trusts' research and development departments (Pennine Acute Trust and Bolton NHS Hospitals Trust) and The University of Manchester.

Support from friends and family will be encouraged throughout the recruitment stage and the programme as it can be influential on women's participation in physical activity [33]. Language will be assessed on a one-to-one basis and translators employed where necessary. Recruitment is being predominantly carried out through the full time research midwives in antenatal clinics. Women are able to opt into the study at anytime after reading the invitation letter and receiving verbal information about the study. A screening sheet will be completed to identify their suitability (based on the exclusion criteria above) and will collect demographic details (e.g. ethnicity, age, marital status, level of education and area deprivation). If the woman is suitable for inclusion she will be asked by the research midwife to sign a consent form and a date for the initial session will be set. At the initial session, the research midwife will explain the details of the 10-week programme, ask them to complete the baseline questionnaire and give them the diary and pedometer. If a woman is not suitable for inclusion or she does not want to attend the lifestyle programme, she will be asked to complete a consent form to say that her screening data and clinical outcome data can be used.

### The 10-week lifestyle programme

The lifestyle programme will run for one and a half hours per week for 10 weeks and is supplementary to standard antenatal care. Women will be invited to the 10-week programme at any stage before 30 weeks gestation to ensure completion of the programme before their delivery.

Given the complexities surrounding the psychological and physical causes of obesity, a multi-component intervention is being used (as suggested by [12]). The programme will be interactive and cover several aspects of their lifestyle (e.g. physical activity, healthy eating and emotional well-being) and their children's well-being (attendance will be encouraged at some groups). Physical activity is an important aspect and will feature each week in the lifestyle programme. The RCOG clinical guidance, following a review of the available evidence around pregnancy and the post-partum period [31], concluded that, in most cases, 'exercise is safe for both mother and fetus during pregnancy.' There are also reports of psychological benefits of exercising for the mother [34,35].

Physical activity is generally low in pregnancy [e.g. [36]] but the reasons for this are not clearly understood. A recent study found that the main barriers (reported in 85% of cases) were intrapersonal (e.g. health-related) [37]. In addition, women are not currently being supplied with the message that physical activity during pregnancy is safe [38] only half of obstetricians discuss physical activity with pregnant women and those who do are likely to advise not to start exercise [39]. Physical activity will be discussed throughout the programme as it needs to be incorporated easily into peoples' everyday lives if they are to adopt it [40]. To achieve this, a trained and insured physical activity instructor will provide half an hour of activity each week in the intervention and will suggest exercises that the women can incorporate into their lifestyle.

### Theoretical framework - Behaviour Change Theory

There are a large number of theoretical approaches to behaviour change concentrated in the health psychology literature [41]. Theory-based interventions and detailed descriptions of the behaviour change techniques used in interventions are essential [42]. However, we must be aware when applying psycholgical theories to behaviour change that the determinants of behaviour change are different to the determinants of behaviour so we must focus on predicting and understanding what needs to change and how we can change it instead of simply describing the behaviour or the intention to change [43]. Our lifestyle programme will use the Social Learning Theory as a tool to encourage behaviour change [27]. Social Learning (cognitive) Theory states that we learn behaviours by cognitive and behavioural processes such as reinforcement of positive and negative behaviour [26]. Social Learning (cognitive) Theory outlines the mechanisms through which determinants influence behaviour and thus states how you can change behaviour using them so is deemed suitable for application. The lifestyle programme will use five components of Social Learning Theory to provide women with an opportunity to gain the skills and knowledge needed to improve their lifestyle; self-efficacy, outcome expectancies (beliefs about the outcomes of an alternative behaviour), goal setting, feedback and positive reinforcement (Table 1 outlines the techniques used in the current lifestyle programme study and the link to theoretical framework).

**Table 1:** Behavioural change techniques used in the 10-week lifestyle programme

Component	Objective	Technique
**Psychological variables**		

Self-efficacy	To increase the women's self-efficacy, so they are more motivated to make healthy changes to their lifestyle	Self-efficacy will be increased by: -Problem solving activities in 10-week programme (e.g. minor ailments in pregnancy pass-the-parcel) -Feedback and reinforcement through the group at the 10-week programme -Personal goal setting during 10-week programme - the setting of a realistic and short-term goal will increase experience of success

Outcome expectations	Develop realistic expectations about the benefits of improving their well-being (physical activity, healthy eating and emotional well-being - relaxation)	When setting their personal goal and in the questionnaires, they will be asked to think about the outcomes of any changes to their behaviour and the impact on their lifestyle. The barriers and facilitators of goal attainment will be discussed at the goal setting stage (week 1 of 10-week programme) and goal attainment stage (week 10 of 10-week programme) Baseline questionnaire and goal setting stage (week 1 of 10-week programme) will ask open questions on their expectations and views regarding behaviour change

Feedback	To provide constant feedback to the women throughout the programme to encourage the uptake of healthy behaviours and changes to their lifestyle	Feedback will take the form of changes to their lifestyle as recorded in their diary and involvement in the lifestyle programme (via health care professionals and other women in the group)

Positive reinforcement	Positive reinforcement will be received by women when they engage in healthy behaviours or changes to their lifestyle	Positive reinforcement will be offered to the women to increase their self-efficacy, this will occur through the other women in the group, the health care professionals and self-monitoring of their diary entries

**Social variables**		

Social Support	Support and advice will be received from health care professional and other pregnant women with a BMI of 30 kg/m^2 ^or more	Receive support and advice from Health Care Professionals at the 10-week programme (fitness instructor, Health Psychologist and midwife) Be offered support from other pregnant women with a BMI of 30 kg/m^2 ^or more at the 10-week programme

**Environmental variables**		

Access to health care	Women will be provided with information about other programmes and services that may be of relevance to them during their pregnancy and in the post-partum stage	Women will be provided with information about other programmes and services that may be of relevance to them during their pregnancy (e.g. one session in the 10-week programme is dedicated to signposting to other services). Additional activities (e.g. aquanatal class, supermarket tour and pampering session) will be offered to the women alongside the 10-week programme as taster sessions.

Self-efficacy is central to Social Learning Theory, unless a person believes that they can produce the desired outcomes through the behaviour they have little reason to act. Self-efficacy regulates motivation and determines the goals people set, their determination to meet their goals and the outcomes they expect. Self-efficacy can be increased by four processes. Firstly, if we experience successes then our self-efficacy rises. Each week the women will take part in small problem-solving activities which they can succeed at (e.g. pass-the-parcel game where women had to decide if pregnancy statements were true facts or myths). Secondly, our self-efficacy rises if we see those similar to ourselves succeed; the women will share experiences as a group so that they can share each others experiences and talk about the reasons for their successes and failure (reinforcement and feedback). The third process is through social persuasion, if we are persuaded verbally that we can do something then we are more likely to believe it. To encourage the women, they will receive educational sessions from health care professionals about their health behaviours and their goals for the programme will be discussed with a health psychologist. Finally, our somatic and emotional state influences our self-efficacy; positive mood enhances self-efficacy and makes us view fatigue more favourably. The women will be in the same group for the whole programme so will gain social support and feedback on their behaviour to provide an environment to foster positive mood. Social support has been shown to have a positive impact on our health [44].

The programme will commence with a one-to-one session with a health psychologist to set a personal goal. This will help women to develop a greater feeling of control (self-efficacy) over their behaviour and health and thus empower the women [45]. As stated above, when people have greater levels of self-efficacy they are more likely to engage in certain behaviours. The goal will be broken down into several smaller goals which are easier for the women to achieve, this will be encouraged by following the principles of SMART (specific, measurable, achievable, relevant and timely). It is important at this stage to explore the women's outcome expectations of the behaviour as we must understand the women's attitudes towards their weight and lifestyle if we are to successfully help them to change this behaviour [46]. Therefore, when setting the goal, the women must be encouraged to think about things that will help them to reach these goals and things that will act as a barrier to achieving their goal. They will also be asked to think about previous experiences and why they failed to reach similar goals in the past. The women will be encouraged to self-regulate their behaviour and the attainment of their goals. One reason for this is that people who reward their own achievements achieve more than those who do the same activities without self-rewards. In addition, feedback is vital for the women to change their behaviour [47]. The women will complete a daily dairy (see data collection section for more information). The diary will also act as a behavioural change technique by self-regulation which in turn increases motivation.

### The post-partum programme

Women are reported to be most likely to return to physical activity 4-6 weeks post-partum [48], therefore, women will be invited to a one-off follow up session at this stage. This session will consist of physical activity such as walking with prams and a focus-group to examine their experiences and views of the programme. If women cannot attend this session, they will be provided with information about post-partum physical activity and an individual interview.

### Data Collection and analysis

Mixed-methods of data collection will be employed (Table 2 outlines the data collection and outcome measures); questionnaires including validated measures [31,49-51] and open questions (given out at baseline, the start of the 10-week programme, the end of the 10-week programme and at follow-up), diaries (completed from baseline to follow-up to provide detailed qualitative data) and focus groups at 4-6 weeks post-partum (interviews will be offered where attendance is not possible). The diaries will provide qualitative data daily insight into the women's feelings during pregnancy, behaviour change and any barriers and facilitators to this behaviour change. In addition, the women's weight gain during pregnancy (women will be weighed at their booking appointment at the end of their pregnancy) and clinical outcome data (e.g. type of birth and blood loss) will be examined. The focus groups will provide follow-up data about the women's views towards the programme after giving birth.

**Table 2:** Data collection and outcome measures

	Recruitment (screening sheet)	Recruitment (baseline)	Start of 10-week programme	End of 10-week programme	10-week programme register	End of pregnancy	Follow-up questionnaire	Follow-up interview/focus group	Diary
Demographics	X								

Physical Activity Readiness Questionnaire (PARQ) - [31]	X	X							

Weight (weight gain during pregnancy and Body Mass Index [BMI])	X					X			

Pregnancy physical activity questionnaire (PPAQ) [49]		X	X	X			X		

Physical activity changes - barriers/facilitators to this change			X	X			X	X	X

Attitudes towards physical activity		X	X	X			X	X	X

Attitudes towards their pregnancy and baby		X	X	X			X	X	X

Food intake changes - barriers/facilitators to this change			X	X			X	X	X

Attitudes towards food intake		X	X	X			X	X	X

General Self-efficacy scale (GSES) [50]		X	X	X			X		

Well-being (W-BQ12) [51]		X	X	X			X		

Attitudes towards their weight			X	X			X		X

Experience of advice received about weight gain during pregnancy	X	X	X	X			X	X	

View of health care and treatment from health care professionals		X	X	X			X	X	X

Views and attitudes towards 10-week programme				X				X	X

Attendance at 10-week programme				X	X			X	X

Outcome expectations of factors influencing pregnancy		X	X	X			X	X	

Outcome expectations of study participation		X					X		

Goal setting			X						

Goal attainment - barriers/facilitators				X			X		X

Clinical outcome data (e.g., mode of birth and blood loss)						X			

To gain a comprehensive view of the programme, qualitative and quantitative data will be combined and interpreted from different vantage points (e.g. demographic differences). Qualitative data will be transcribed verbatim and analysed using an interpretive approach. Thematic analysis will be used as the process organises and minimises the data whilst maintaining the detail as it '...is a method for identifying, analysing and reporting patterns (themes) within data' (p.79 [52]). It can be applied both in a deductive and inductive nature, thus suitable for extracting new themes from the data as required in a feasibility study with little evidence-base. In addition, thematic analysis allows for theme extraction for each woman individually (vertical analysis) and also between groups (e.g. horizontal analysis of subgroups such as demographics or 10-week programme cohort) resulting in rich results.

Quantitative data will be input onto Statistical Package for Social Scientists (SPSS). Much of the quantitative analysis will be descriptive in nature; findings will be displayed as frequencies and Chi-squared analysis used to explore associations in the data. Pre and post programme data will be examined using Chi-squared and t-tests analysis. The necessary data will be collected from all participants (e.g. the 10-week programme cohort and number of sessions attended) to allow for multi-level analysis taking into account the effect of group dynamics if required.

## Discussion

This study protocol paper includes detail and description of the rationale and methodology used to design the community lifestyle programme and the feasibility study to evaluate this programme in pregnant women with a BMI of 30 kg/m^2 ^or more. When designing an effective intervention you must start by understanding the views and attitudes of the recipients towards the behaviours the intervention targets [53]. Therefore, the current mixed-methods study will explore the ways in which weight and pregnancy are embodied and experienced within the lives of women in England. Findings from this feasibility study will inform future interventions and NHS services and add to the evidence base as concluded a necessity by NICE (2009). Descriptive data will be collected outlining the pregnancy and health care experience of women with a BMI of 30 kg/m^2 ^or mores' experiences of pregnancy and health care during pregnancy. The feasibility of a community lifestyle programme with pregnant women with a BMI of 30 kg/m^2 ^or more will be determined. More specifically, findings will indicate which aspects of the intervention are acceptable and suitable to pregnant women with a BMI or 30 kg/m^2 ^or more. Techniques to encourage behaviour change with this target group will also be highlighted, and detail provided regarding the suitability of social learning theory as a behaviour change tool. As previously mentioned obesity is high on the Governments' agenda and as a consequence there is likely to be a plethora of intervention studies aimed at reducing maternal weight. This study will aid in the design of such work. A multi-disciplinary dissemination plan has been devised to ensure that the findings of this feasibility study reach a wide audience and can be used to design future lifestyle programmes with the same or other populations.

## List of abbreviations

Abbreviations are defined in the text when they first appear and not listed here.

## Competing interests

The authors declare that they have no competing interests.

## Authors' contributions

DS is responsible for coordinating the study, contributed to the formulation of the study design and drafted the manuscript.

MW is on the advisory board of this study and contributed to the formulation of the study design.

CS is one of the co-applicants of this study and contributed to the formulation of the study design.

WT is responsible for data collection and the delivery of the intervention protocol outlined in this paper.

JG is responsible for data collection and the delivery of the intervention protocol outlined in this paper.

CC is responsible for data collection and the delivery of the intervention protocol outlined in this paper.

TL led on the formulation of the study design and is responsible for supervising this project.

All authors read and commented on drafts of the manuscript and agreed on the final version.

## Authors' information

Debbie Smith, PhD, CPsychol, MSc, BSc (Hons) - Research Associate, The School of Nursing, Midwifery and Social Work, The University of Manchester, UK.

Melissa Whitworth, BSc (Hons), MBChB (Hons), MRCOG, MD - Clinical Lecturer in Obstetrics and Gynaecology, Department of Obstetrics, St Mary's Hospital, Manchester, UK

Colin Sibley, PhD, BSc - Professor of Child Health and Physiology, Director Tommy's the baby charity Maternal and Fetal Health Research Group, Manchester, UK.

Wendy Taylor, RM - Research Midwife, The School of Nursing, Midwifery and Social Work, The University of Manchester, UK.

Jane Gething RM, RGN, BA (hons) - Research Midwife, The School of Nursing, Midwifery and Social Work, The University of Manchester, UK.

Catherine Chmiel RM - Research Midwife, The School of Nursing, Midwifery and Social Work, The University of Manchester, UK.

Tina Lavender, PhD, MSc, RM - Professor of Midwifery, The School of Nursing, Midwifery and Social Work, The University of Manchester, UK.

## Pre-publication history

The pre-publication history for this paper can be accessed here:

http://www.biomedcentral.com/1471-2458/10/284/prepub

## References

[B1] World Health Organization Obesity. Preventing and managing the global epidemic. Report of a WHO consultation on obesity WHO/NUT/NCD/981, WHO, Geneva 1999 11234459

[B2] Centres for Disease control & Prevention Obesity and Overweight factsheet http://www.cdc.gov/nchs/fastats/overwt.htm Retrieved November 23^rd ^2009

[B3] Avlott J Brown I Copeland R Johnson D Tackling obesities: The foresight report and the implications for local Government 2008 Sheffield Hallam University

[B4] Bowers D Cohen W Obesity related pregnancy complications in an inner-city clinic Journal of Perinatology 1999 19 216 219 10.1038/sj.jp.7200143 10685225

[B5] Catalano PM Ehrenberg HM The short and long-term implications of maternal obesity on the mother and her offspring British Journal of Obstetrics and Gynaecology 2006 113 1126 1133 16827826 10.1111/j.1471-0528.2006.00989.x

[B6] Sebire NJ Jolly M Harris JP Wadsworth J Joffe M Beard RW Regan L Robinson S Maternal obesity and pregnancy outcome: a study of 287 213 pregnancies in London International Journal of Obesity 2001 25 1175 1182 10.1038/sj.ijo.0801670 11477502

[B7] Cedergren M Kallen B Maternal obesity and the risk for orofacial clefts in the offspring Cleft Palate Craniofac Journal 2005 42 367 371 10.1597/04-012.1 16001917

[B8] Weiss R Dziura J Burgert TS Obesity and the metabolic syndrome in children and adolescents N Engl J Med 2004 350 2362 2374 10.1056/NEJMoa031049 15175438

[B9] CDC 2007 pregnancy nutrition surveillance. Nation. Summary of trends in maternal health indicators http://www.cdc.gov/PEDNSS/pnss_tables/html/pnss_national_table16.htm Retrieved on November 18^th ^2009

[B10] Heselhurst N Lang R Rankin J Wilkinson J Summerbell C Obesity in pregnancy; A study of the impact of maternal obesity on NHS maternity services British Journal of Obstetrics and Gynaecology 2007 114 334 342 17261124 10.1111/j.1471-0528.2006.01230.x

[B11] Lewis G (ed) The Confidential Enquiry into Maternal and Child Health (CEMACH) Saving Mothers' Lives: reviewing maternal deaths to make motherhood safer - 2003-2005. The Seventh Report on Confidential Enquiries into Maternal Deaths in the United Kingdom 2007 London: CEMACH

[B12] Dodd JM Crowther CA Robinson JS Dietary lifestyle interventions to limit weight gain during pregnancy for obese or overweight women: A systematic review Acta. Obestetricia et Gynecologica 2008 87 702 706 10.1080/00016340802061111 18607830

[B13] Institute of Medicine Weight gain during pregnancy: Reassessing the guidelines 2009 Washington, DC: National Academy Press

[B14] Cedergren M Effects of gestational weight gain and body mass index in obstetric outcome in Sweden International Journal of Gynecology & obstetrics 2006 93 269 274 10.1016/j.ijgo.2006.03.002 16626716

[B15] Sattar N Greer IA Pregnancy complications and maternal cardiovascular risk: opportunities for intervention and screening? BMJ 2002 325 157 60 10.1136/bmj.325.7356.157 12130616 PMC1123678

[B16] National Collaborating Centre for Women's and Children's Health Antenatal care: routine care for the healthy pregnant woman (Clinical guideline; no. 62) 2008 London (UK): National Institute for Health and Clinical Excellence (NICE) 56

[B17] Bell R Weir Z Stothard K Pearce M Adamson A "Maternal obesity: when should we intervene? A systematic review of lifestyle interventions in women before, during and after pregnancy" International Journal of Obesity 2007 31 S40

[B18] Campbell F Messina J Johnson M Guillaume L Madan J Goyder E Systematic review of dietary and/or physical activity interventions for weight management in pregnancy 2009 The University of Sheffield: ScHARR Public Health Collaboration Centre

[B19] Messina J Johnson M Campbell F Everson Hock E Guillaume L Duenas A Rawdin A Goyder E Chilcott J Systematic review of weight management interventions after childbirth 2009 The University of Sheffield: ScHARR Public Health Collaboration Centre

[B20] Dixon A Motivation and confidence: What does it take to change behaviour? 2008 London: The Kings Fund

[B21] Krishnamoorthy U Schram CMH Hill SR Maternal obesity in pregnancy: Is it time for meaningful research to inform preventive and management strategies? British Journal of Obstetrics & Gynaecology 2006 113 1134 1140 10.1111/j.1471-0528.2006.01045.x 16972858

[B22] Smith SA Hulsey T Goodnight W Effects of obesity on pregnancy JOGNN 2008 37 176 184 18336441 10.1111/j.1552-6909.2008.00222.x

[B23] De Vault M Feeding the Family: the Social Organisation of Caring as Gendered Work 1991 Chicago: University of Chicago Press

[B24] Lupton D Food, the Body and the Self 1996 London: Sage

[B25] Anderson RE Wadden TA Bartlett SJ Zemel B Verde TJ Franckowiak SC Effects of lifestyle activity vs structured aerobic exercise in obese women Journal of the American Medical Association 1999 281 4 335 340 10.1001/jama.281.4.335 9929086

[B26] Bandura A Social foundations of thought and action: A social cognitive theory 1986 Englewood Cliffs, NJ: Prentice-Hall

[B27] Elder JP Ayala GX Harris S Theories and intervention approaches to health-behaviour change in primary care American Journal of Preventive Medicine 1999 17 4 275 284 10.1016/S0749-3797(99)00094-X 10606196

[B28] Rootman I Goodstadt M Hyndman B McQueen DV Potvin L Springett J Ziglio E Evaluation in health promotion: principles and perspectives 2001 Copenhagen; WHO Regional Office for Europe 11729787

[B29] Medical Research Council A framework for development and evaluation of RCTs for complex interventions to improve 2000 http://www.mrc.ac.uk/Utilities/Documentrecord/index.htm?d=MRC003372 Retrieved on 7^th ^April 2010

[B30] Department of Health Health Profiles 2008 Association of Public Health Observatories http://www.healthprofiles.info Retrieved on November 11^th ^2009

[B31] Thomas S Reading J Shepard RJ Revision of the physical activity readiness questionnaire Canadian Journal of Sport Science 1992 17 338 45 1330274

[B32] Royal College of Obstetricians and Gynaecologists Exercise in pregnancy 2006 London: Royal College of Obstetricians and Gynaecologists

[B33] Symons Downs D Hausenblas HA Women's exercise beliefs and behaviours during their pregnancy and postpartum Journal of Midwifery & Women's Health 2004 49 2 138 144 10.1016/S1526-9523(03)00495-1 15010667

[B34] Armstrong K Edwards H The effectiveness of a pram-walking exercise programme n reducing depressive symptomatology for postnatal women International Journal of Nursing Practice 2004 10 177 194 10.1111/j.1440-172X.2004.00478.x 15265228

[B35] Paisley TS Joy EA Price RJ Exercise during pregnancy: A practical approach Current Sports Medicine Reports 2003 2 325 30 14583162 10.1249/00149619-200312000-00008

[B36] Fell DB Joseph KS Armson A Dodds L The impact of pregnancy on physical activity level Mat Child Health J 2009 13 359 603 10.1007/s10995-008-0404-7 18719984

[B37] Evenson KR Moss MK Carrier K Siega-Riz AM Perceived barriers to physical activity among pregnant women Maternal Child Health Journal 2009 13 364 375 10.1007/s10995-008-0359-8 PMC2657195 18478322

[B38] Clarke PE Gross H Women's behaviour, beliefs and information sources about physical exercise in pregnancy Midwifery 2004 20 133 141 10.1016/j.midw.2003.11.003 15177856

[B39] Entin PL Munhall KM Recommendations regarding exercise during pregnancy made by private/small group practice obstetricians in the USA Journal of Sports Science & Medicine 2006 5 449 458 PMC3842146 24353463

[B40] Drummond S Obesity in the UK Community Practitioner 2003 76 3 79 80

[B41] Abrahams C Michie S A taxonomy of behaviour change techniques used in interventions Health Psychology 2008 27 3 379 387 10.1037/0278-6133.27.3.379 18624603

[B42] Michie S Abrahams C Interventions to change health behaviours: evidence-based or evidence-inspired? Psychology & Health 2004 19 1 29 49 10.1080/0887044031000141199

[B43] Brug J Oenema A Ferreira I International Journal of Behavioural Nutrition and Physical Activity 2005 2 2 9 10.1186/1479-5868-2-2 PMC1087867 15807898

[B44] Berkman LF Syme SL Social networks, host resistance, and mortality: A nine year follow-up study of Alameda County residents American journal of Epidemiology 1979 109 2 186 204 425958 10.1093/oxfordjournals.aje.a112674

[B45] Bandura A Health promotion from the perspective of the Social Cognition Theory Psychology & Health 1998 13 623 649 10.1080/08870449808407422

[B46] Gray-Donald K Robinson E Collier A David K Renaud L Rodrigues S Intervening to reduce weight gain in pregnancy and gestational diabetes mellitus in Cree communities: an evaluation Canadian Medical Association Journal 2000 163 10 1247 1251 11107459 PMC80308

[B47] Claesson I-M Josefsson A Cedergren M Brynhildsen J Jeppsson A Nystrom F Sydsjo A Sydsjo G Consumer satisfaction with a weight-gain intervention programme for obese pregnant women Midwifery 2008 24 163 167 10.1016/j.midw.2006.10.007 17316933

[B48] ACOG Committee on Obstetric Practice ACOG Committee opinion. Number 267, January 2002: exercise during pregnancy and the postpartum period Obstetrics and Gynecology 2002 99 1 171 3 10.1016/S0029-7844(01)01749-5 11777528

[B49] Chasen-Taber L Development and validation of a pregnancy physical activity questionnaire Medicine & Science in Sports & Exercise 2004 36 1750 1760 10.1249/01.MSS.0000142303.49306.0D 15595297

[B50] Chen G Gully SM Eden D Validation of a New General Self-Efficacy Scale Organizational Research Methods 2001 4 1 62 68 10.1177/109442810141004

[B51] Bradley C Bradley C The well-being question Handbook of psychology and diabetes: A guide to psychological measurement in diabetes research and practice 1994 Chur, Switzerland: Harwood Academic Publishers

[B52] Braun V Clarke V Using thematic analysis in psychology Qualitative Research in Psychology 2006 3 77 101 10.1191/1478088706qp063oa

[B53] Symons Downs D Hausenblas HA Women's exercise beliefs and behaviours during their pregnancy and postpartum Journal of Midwifery & Women's Health 2004 49 2 138 144 10.1016/S1526-9523(03)00495-1 15010667

